# The safety of sotagliflozin in the therapy of diabetes mellitus type 1 and type 2: A meta-analysis of randomized trials

**DOI:** 10.3389/fendo.2022.968478

**Published:** 2022-09-26

**Authors:** Feifei Zhou, Nannan Du, Lulin Zhou, Chenxi Wang, He Ren, Qiang Sun

**Affiliations:** ^1^ Laboratory of Cell Engineering, Institute of Biotechnology, Research Unit of Cell Death Mechanism, Chinese Academy of Medical Science, Beijing, China; ^2^ The Second Clinical Medical College, Zhejiang Chinese Medical University, Hangzhou, China

**Keywords:** Sodium-glucose cotransporter 1 (SGLT1), sodium-glucose cotransporter 2 (SGLT2), sotagliflozin, safety, diabetes mellitus, meta-analysis

## Abstract

**Background:**

Diabetes mellitus (DM) is a global health problem, and it has become a shocking threat in the contemporary era. The objective of this study was to analyze the safety of sotagliflozin in patients with DM systematically and intuitively.

**Methods:**

On November 15, 2021, literature retrieval was performed on PubMed, Web of Science, EBSCO, and Cochrane libraries. The meta-analysis results included genital mycotic infection, related-to-acidosis events, and other related adverse events, including diarrhea, severe nocturnal hypoglycemia event, and volume depletion. In addition, a subgroup analysis was also conducted based on different doses of sotagliflozin. Moreover, the patient-treated years analyzed in the study were 12 weeks, 24 weeks, and 52 weeks, respectively, for type 1 diabetes, and were 12 weeks, 22 weeks, and 52 weeks, respectively, for type 2 diabetes.

**Results:**

The results of this meta-analysis illustrated that sotagliflozin could increase the risk of genital mycotic infection for patients with T1D and T2D (RR: 3.49, 95% Cl: 2.54-4.79, *p* < 0.001; RR: 2.83, 95% Cl: 2.04-3.93, *p* < 0.001; respectively). In addition, the subgroup analysis showed that the drug doses that could increase the risk of genital mycotic infection were 400 mg and 200 mg (RR: 3.63, 95% Cl: 2.46-5.36, *p* < 0.001; RR: 3.21, 95% Cl: 1.84-5.62, *p* < 0.001; respectively) in T1D. Moreover, sotagliflozin could increase the risk of events related to acidosis in the patients of T1D, including acidosis-related adverse events, positively adjudicated diabetic ketoacidosis, acidosis-related event, and diabetic ketoacidosis (RR: 7.49, 95% Cl: 3.20-17.52, *p* < 0.001; RR: 6.05, 95% Cl: 2.56-14.30, *p* < 0.001; RR: 4.83, 95% Cl: 3.13-7.45, *p* < 0.001; RR: 8.12, 95% Cl: 3.06-21.52, p < 0.001; respectively). In the patients of T2D, sotagliflozin could not increase the risk of DKA (RR: 1.30, 95% Cl: 0.34-4.99, p = 0.70). About serious of acidosis-related adverse events, positively adjudicated diabetic ketoacidosis (DKA) and acidosis-related event, the included studies were not reported for T2D patients. As for the other related adverse events, sotagliflozin was found to be a risk factor for diarrhea and volume depletion in T1D patients (RR: 1.44, 95% Cl: 1.09-1.90, p = 0.01; RR: 2.50, 95% Cl: 1.33-4.69, p < 0.01; respectively) and T2D patients (RR: 1.44, 95% Cl: 1.26-1.64, p < 0.001; RR: 1.25, 95% Cl: 1.07-1.45, p < 0.01; respectively).

**Conclusions:**

This meta-analysis showed that the adverse events of sotagliflozin were tolerable to patients with DM, in terms of the incidence of genital mycotic infection, related-to-acidosis events, diarrhea, volume depletion, and severe nocturnal hypoglycemia events. In addition, the subgroup analysis of sotagliflozin dosage is considered to have great clinical significance for future guidance of sotagliflozin application in patients with DM.

## Introduction

Diabetes mellitus (DM) has always been a global health problem, and it has contemporarily become an increasingly alarming threat. According to the latest statistics report in 2020, DM is more common in developed countries than the developing countries. The number of DM patients is gradually increasing at a dramatic rate, and soon it may affect every household and even everyone in the family (1). There is therefore an urgent need to develop new entities that can meet the current scarcity of anti-diabetic drugs. Due to the unique mechanism of sodium-glucose cotransporter 1 (SGLT1) and sodium-glucose cotransporter 2 (SGLT2), which is independent of the insulin signaling pathway, they are regarded as prominent targets for the treatment of DM.

SGLT1 is functional in the gastrointestinal tract as the primary transporter for the absorption of glucose and galactose. SGLT2 is expressed in the kidney and could reabsorb 90% of the filtered glucose (2). Since insulin therapy alone cannot achieve adequate control of blood sugar in most DM patients (3), sotagliflozin, a dual inhibitor that targets SGLT1 and SGLT2 (4), was currently approved in Europe as the best auxiliary drug with insulin for the treatment of adults with DM and a body mass index (BMI) of ≥ 27 kg/m^2^ (5). The primary metabolic effect of sotagliflozin is mediated by SGLT2-inhibition in the kidney, while some additional postprandial glucose control may be mediated by inhibition of SGLT1 in the intestine (3, 4, 6–9). This dual inhibitory effect of sotagliflozin can delay and inactivate intestinal glucose absorption after meals. Sotagliflozin can prolong the local inhibitory effect of SGLT1 in the intestine in the aspect of absorption to ≥ 5 hours, resulting in an additional reduction in intestinal glucose absorption. The extended absorption can further/subsequently enhance the efficacy of SGLT2 inhibitors and ultimately reduce PPG and insulin levels (5, 10); sotagliflozin can also lead to an increase in renal glucose excretion and a decrease in the early-stage glucose absorption, as well as an increase in GLP-1 and PYY (11). This is different from the SGLT2-selective inhibitors, such as dapagliflozin, empagliflozin, and ertugliflozin, which have a prominent role nowadays in the treatment of type 2 diabetes (T2D), cardiovascular and renal disease (especially dapagliflozin and empagliflozin). Canagliflozin is also said to exhibit some SGLT1-inhibition, but the extent is not precisely quantified.

However, as a dual inhibitor that targets both SGLT1 and SGLT2, sotagliflozin is not available as a “marketed” pharmaceutical compound worldwide, the adverse events of sotagliflozin treatment for DM patients, such as genital infections, DKA, diarrhea, and increased volume loss, could not be neglected (8, 10, 12). Therefore, this study is an attempt to systematically and intuitively analyze the safety of sotagliflozin in DM treatment.

## Methods

### Search strategy

From the beginning of retrieval to November 15, 2021, this meta-analysis searched PubMed, Web of Science, EBSCO, and Cochrane libraries for relevant research. The combination of free-text terms and medical subject headings was used in this meta-analysis. Terms that were used for searching include “sotagliflozin”, “LX4211”, “LX-4211”, “diabetes mellitus”, “diabetic Mellitus”, “diabetes”, “diabetic”, “experimental diabetic”, “type 2 diabetes mellitus”, “II diabetes”, “type 2 diabetes”, “type 1 diabetes mellitus”, “I diabetes”, and “type 1 diabetes”. In addition, we manually reviewed the references in the relevant literature.

### Inclusion and exclusion criteria

Inclusion criteria: (1) The included articles were English reports of completed clinical randomized controlled trials that focused on the efficacy and safety of sotagliflozin in DM treatment, (2) the included articles were RCTs with sotagliflozin involved in phases II or III; (3) the included articles mentioned the related adverse events.

Exclusion criteria: (1) Study contained inappropriate subgroup analysis or single-arm treatment; (2) the research was not a phase II or phase III clinical trial; (3) the adverse events were absent in the article; (4) the research data was not able to be extracted; (5) The latest article will be selected and reviewed when it comes to duplicates.

### Outcome measures

The results of this study were genital mycotic infection, related-to-acidosis events, and other related adverse events, including diarrhea, severe nocturnal hypoglycemia event, volume depletion, and the like. In addition, we also conducted a subgroup analysis based on different doses of sotagliflozin.

### Assessment of the risks of bias and data extraction

We assessed the potential risks of bias in trials by using the Cochrane Collaboration Risk of Bias Assessment tool (13), which includes the aspects of sequence generation, allocation concealment, blinding, incomplete outcome data, selective outcome reporting, and free of other bias (**Supplementary Table 2**). Two investigators (A.A. and N.A.) completed the review independently. Disagreements were resolved by a third investigator (I.A.). Two researchers independently extracted the basic information of each study. Baseline information included: author, publication year, country, the medication regimen of the experimental group and the control group, number of participants in the study, and the adverse events.

### Statistical analysis

We compared the adverse events in the sotagliflozin group versus the control groups, expressed with risk ratios (RR), and the confidence interval was set to 95% credible interval (95%Cl). The subgroup analyses of the adverse events were also expressed with RR and a 95% confidence interval. As for the heterogeneity, this meta-analysis selected the fixed-effects and random-effects models according to the size of I^2^ to increase reliability, that is, when I^2^ > 50%, a random effects model was used, and when I^2^ ≤ 50%, a fixed effects model was used. The study also evaluated the publication bias by employing the Begg’s and Egger’s tests, p < 0.05 would be considered to be statistically significant. The Reviewer Manager 5.4 Software was used to evaluate the adverse events, and the Stata 15.1 Software was used to evaluate the sensitivity bias (14).

## Results

### Eligible studies and inclusion characteristics

A total of 217 clinical trials from the database were initially retrieved based on the search strategy mentioned, among which three studies were manually retrieved. Nine clinical studies (3, 8, 9, 15–20) were finally selected (including a total number of 15,519 patients with T1D and T2D diagnoses who were male or female, 3203 T1D patients and 12316 T2D patients, respectively) after the final screening and qualification assessment. The stringent screening criteria ruled out articles with inappropriate subgroup analysis or single-arm studies, non-phase II or phase III clinical trials, those with unextractable data, and those for secondary analysis. The detailed search and screening process is shown in **Supplementary Figure 1**. Throughout the nine included studies with 15,519 patients involved, five of the studies (3, 8, 9, 15, 18) had set their subjects as patients with T1D and four (16, 17, 19, 20) were patients with T2D. The nine studies were RCTs, including two phase II and seven phase III clinical trials. The experimental groups involved were patients receiving sotagliflozin 75 mg, 200 mg and 400 mg (n = 2), sotagliflozin 200 mg and 400 mg (n = 3), sotagliflozin 400 mg (n = 2), sotagliflozin 200 mg or 400 mg (n = 2), and the control groups were given placebo. About the number of patient-treated years, there were 12 weeks (Baker et al., 2019; Bode et al., 2021), 24 weeks (Garg et al., 2017), and 52 weeks (Buse et al., 2018; Danne et al., 2018) in T1D, 12 weeks (Rosenstock et al., 2015), 22 weeks (Bhatt et al., 2021; Bhatt et al., 2021*) because of loss of funding from the sponsor, and 52 weeks (Cherney et al., 2021) in T2D, respectively (“*” indicated that it was not the same study). The included research features are detailed in **Supplementary Table 1**.

### The effect of sotagliflozin on genital mycotic infection

**Figure 1:**
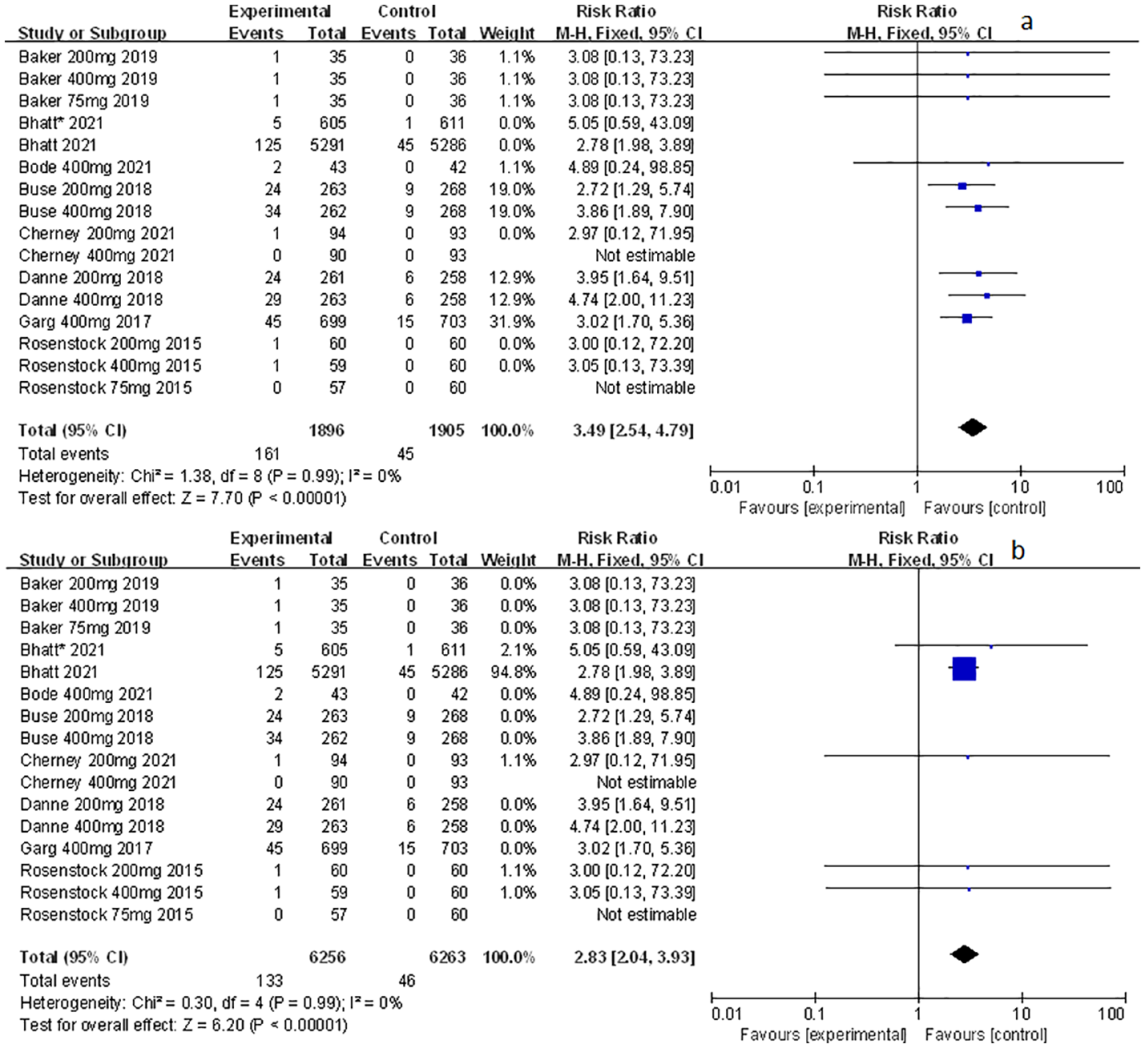
Forest plots of genital mycotic infection for sotagliflozin versus the control group in T1D and T2D. **(A)** The adverse events of genital mycotic infection in T1D. **(B)** The adverse events of genital mycotic infection in T2D.

**Figure 2:**
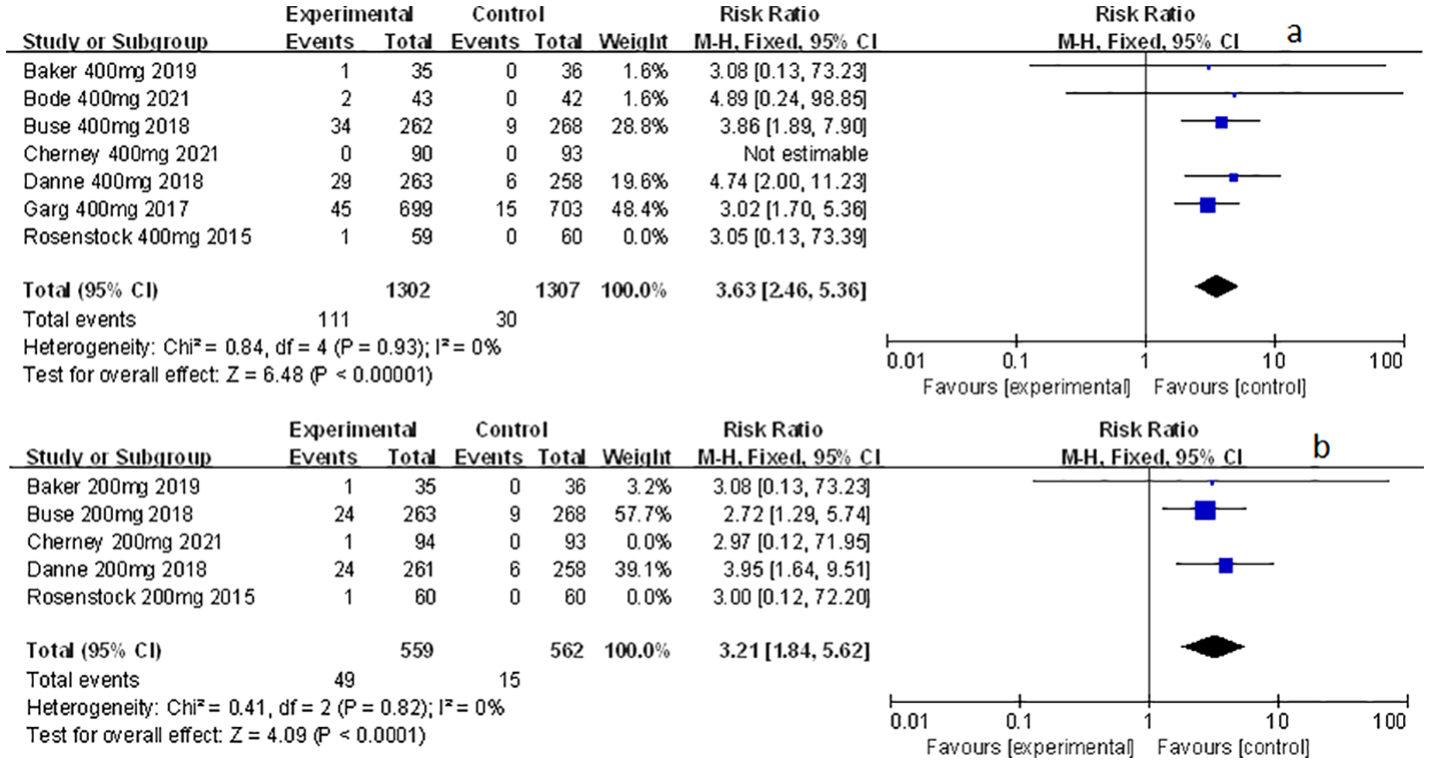
Forest plot of genital mycotic infection in the subgroup of sotagliflozin 400mg and 200mg versus the control group in T1D. **(A)** The adverse events of genital mycotic infection in the subgroup of sotagliflozin 400mg versus placebo. **(B)** The adverse events of genital mycotic infection in the subgroup of sotagliflozin 200mg versus placebo.

Among the included studies, nine of them involved genital mycotic infection, with a population of 15,519 patients covering, of which 3,203 were T1D patients and 12,316 were T2D patients. The data displayed in the study allowed for overall analysis. Genital mycotic infection was used as the event variable to reveal that sotagliflozin was a related risk factor for patients with T1D and T2D (RR: 3.49, 95% Cl: 2.54-4.79, *p* < 0.001; **Figure 1A**; RR: 2.83, 95% Cl: 2.04-3.93, *p* < 0.001; **Figure 1B**; respectively), the heterogeneity was 0. In addition, a subgroup analysis against a dosage of sotagliflozin was conducted which illustrated that sotagliflozin could increase the risk of genital mycotic infection in the case of applying sotagliflozin 400 mg versus placebo (RR: 3.63, 95% Cl: 2.46-5.36, *p* < 0.001; **Figure 2A**) in T1D, the heterogeneity was 0. In the subgroup of 200 mg sotagliflozin versus placebo, the former was more likely to lead to an increased probability of genital mycotic infection (RR: 3.21, 95% Cl: 1.84-5.62, *p* < 0.001; **Figure 2B**) with a heterogeneity of 0 in T1D. Please see the related adverse events about sotagliflozin in **Table 1**, sotagliflozin 400 mg in **Table 2**, and sotagliflozin 200 mg in **Table 3**.

**Table 1:** Subgroup analysis of the adverse events (AEs).

Experimental vs. control	No. ofstudi es	RR	95%CI	*p*	Heterogeneity	Effect-model
					(I^2^) (%)	
Severe of Acidosis-related adverse events	2	7.49	3.20-17.52	**<0.001**	0	Fixed
Positively adjudicated DKA	4	6.05	2.56-14.30	**<0.001**	0	Fixed
Acidosis-related event	3	4.83	3.13-7.45	**<0.001**	0	Fixed
Amputation	3	4.33	0.74-25.44	0.11	0	Fixed
DKA	6	3.23	1.14-9.19	**0.03**	53	Random
Genital mycotic infection	9	3.16	2.51-3.97	**<0.001**	0	Fixed
Vulvovaginal candidiasis	2	3.03	0.32-28.94	0.33	0	Fixed
Blood ketone body increased	3	2.75	0.97-7.78	0.06	0	Fixed
Vulvovaginal events	3	2.01	0.61-6.67	0.25	0	Fixed
Any event of special interest leading to discontinuation	3	1.94	1.27-2.98	**<0.01**	10	Fixed
Nasopharyngitis	2	1.86	0.77-4.52	0.17	0	Fixed
Diarrhea of Any event of special interest leading to discontinuation	3	1.80	0.60-5.38	0.29	0	Fixed
Genital mycotic infection of Any event of special interest leading to discontinuation	2	1.65	0.52-5.28	0.40	0	Fixed
Diarrhoea	9	1.44	1.28-1.62	**<0.001**	0	Fixed
AEs leading to treatment discontinuation	6	1.40	1.04-1.89	**0.03**	27	Random
Coronary revascularization	3	1.34	0.47-3.87	0.59	0	Fixed
Volume depletion	7	1.31	0.99-1.74	0.06	21	Random
Urinary tract infection of Any event of special interest leading to discontinuation	3	1.29	0.32-5.21	0.72	19	Fixed
Nausea	2	1.27	0.61-2.65	0.52	0	Fixed
Severe AE	5	1.27	0.84-1.93	0.26	33	Random
Myocardial infarction or hospitalization for unstable angina	4	1.26	0.50-3.20	0.62	0	Fixed
Severe AE	7	1.18	0.97-1.45	0.10	29	Random
Malignancy	8	1.15	0.80-1.64	0.46	0	Fixed
Drug-induced liver injury	5	1.13	0.44-2.90	0.80	5	Fixed
Severe treatment-related AE	2	1.12	0.60-2.06	0.73	0	Fixed
Urinary tract infection	9	1.03	0.93-1.13	0.60	0	Fixed
Any AE	8	1.02	0.99-1.06	0.19	0	Fixed
Positively of Hypoglycaemia	5	1.02	0.99-1.05	0.12	0	Fixed
Any of Events of special interest	4	1.00	0.99-1.01	0.73	0	Fixed
Any SMBG value #3.0 mmol/L (#55 mg/dL)	4	1.00	0.96-1.03	0.91	44	Random
Documented (SMBG ≤ 70 mg/dL) of Hypoglycaemia	4	0.99	0.96-1.02	0.42	51	Random
Any nocturnal documented hypoglycemia	3	0.97	0.94-1.00	0.06	0	Fixed
Renal event	6	0.95	0.73-1.24	0.71	0	Fixed
Severe hypoglycemia	7	0.95	0.66-1.38	0.80	37	Random
Bone fracture	8	0.88	0.72-1.09	0.25	0	Fixed
Deaths	7	0.83	0.60-1.15	0.26	0	Fixed
Heart failure hospitalization	4	0.67	0.19-2.35	0.54	0	Fixed
Pancreatitis	6	0.58	0.30-1.13	0.11	0	Fixed
Severe nocturnal hypoglycemia event	3	0.55	0.31-1.00	0.05	0	Fixed
Cardiovascular events	3	0.53	0.26-1.07	0.08	49	Random
Stroke	4	0.41	0.12-1.43	0.16	0	Fixed
Venous thrombotic events	4	0.35	0.03-3.93	0.39	67	Random

The meaning of the bold values are statistically significant.

**Table 2:** Subgroup analysis of the adverse events (AEs) in sotagliflozin 400mg.

Experimental vs. control	No. ofstudies	RR	95%CI	*p*	Heterogeneity	Effect- model
					(I^2^) (%)	
Severe of Acidosis-related adverse events	2	6.96	1.65-29.42	**<0.01**	28	Random
Positively adjudicated DKA	4	5.76	2.34-14.21	**<0.001**	0	Fixed
DKA	4	4.94	0.53-45.99	0.16	53	Random
Acidosis-related event	3	4.59	2.34-8.97	**<0.001**	20	Random
Genital mycotic infection	7	3.62	2.46-5.33	**<0.001**	0	Fixed
Vulvovaginal candidiasis	2	3.03	0.32-28.94	0.33	0	Fixed
Vulvovaginal events	3	3.02	0.61-14.85	0.17	0	Fixed
Blood ketone body increased	3	2.71	0.90-8.15	0.08	0	Fixed
Diarrhea of Any event of special interest leading to discontinuation	3	2.60	0.61-11.18	0.20	0	Fixed
Any event of special interest leading to discontinuation	3	2.51	1.46-4.31	**<0.001**	0	Fixed
Genital mycotic infection of Any event of special interest leading to discontinuation	2	1.78	0.38-8.30	0.46	0	Fixed
AEs leading to treatment discontinuation	5	1.69	1.06-2.72	**0.03**	40	Random
Diarrhoea	7	1.67	1.20-2.32	**<0.01**	0	Fixed
Severe AE	6	1.44	1.10-1.87	**<0.01**	7	Fixed
Nasopharyngitis	2	1.38	0.46-4.14	0.56	0	Fixed
Malignancy	6	1.35	0.47-3.86	0.58	0	Fixed
Severe AE	5	1.35	0.68-2.70	0.39	59	Random
Volume depletion	5	1.28	0.38-4.29	0.69	47	Random
Myocardial infarction or hospitalization for unstable angina	4	1.24	0.14-11.34	0.85	36	Random
Renal event	5	1.06	0.61-1.86	0.83	0	Fixed
Any AE	7	1.02	0.97-1.08	0.40	13	Fixed
Nausea	2	1.02	0.18-5.83	0.99	48	Random
Any of Events of special interest	4	1.01	0.99-1.02	0.56	0	Fixed
Coronary revascularization	3	1.01	0.23-4.44	0.99	0	Fixed
Positively of Hypoglycaemia	4	1.01	0.99-1.03	0.45	0	Fixed
Documented (SMBG ≤ 70 mg/dL) of Hypoglycaemia	4	0.99	0.94-1.05	0.76	38	Random
Any SMBG value #3.0 mmol/L (#55 mg/dL)	4	0.99	0.93-1.04	0.66	61	Random
Any nocturnal documented hypoglycemia	3	0.97	0.93-1.01	0.11	0	Fixed
Cardiovascular events	2	0.90	0.07-11.51	0.94	63	Random
Severe hypoglycemia	5	0.88	0.58-1.33	0.54	10	Fixed
Urinary tract infection	7	0.85	0.62-1.16	0.30	0	Fixed
Drug-induced liver injury	5	0.72	0.10-5.24	0.74	20	Random
Bone fracture	6	0.55	0.30-1.02	0.06	0	Fixed
Stroke	4	0.51	0.09-2.75	0.43	0	Fixed
Deaths	6	0.48	0.16-1.46	0.20	0	Fixed
Severe nocturnal hypoglycemia event	3	0.34	0.13-0.84	**0.02**	0	Fixed

The meaning of the bold values are statistically significant.

**Table 3:** Subgroup analysis of the adverse events (AEs) in sotagliflozin 200mg.

Experimental vs. control	No. ofstudies	RR	95%CI	*p*	Heterogeneity	Effect- model
					(I^2^) (%)	
DKA	3	10.41	1.95-55.43	**<0.01**	0	Fixed
Acidosis-related event	2	6.95	2.29-21.07	**<0.001**	0	Fixed
Amputation	3	4.95	0.58-42.00	0.14	0	Fixed
Urinary tract infection of Any event of special interest leading to discontinuation	2	4.01	0.45-35.80	0.21	0	Fixed
Genital mycotic infection	5	3.20	1.86-5.50	**<0.001**	0	Fixed
Volume depletion	3	2.23	1.02-4.84	**0.04**	0	Fixed
Coronary revascularization	2	1.81	0.39-8.51	0.45	0	Fixed
Myocardial infarction or hospitalization for unstable angina	3	1.59	0.42-6.04	0.50	0	Fixed
Drug-induced liver injury	3	1.55	0.41-5.84	0.51	18	Fixed
Severe hypoglycemia	3	1.37	0.16-11.71	0.77	58	Random
Malignancy	4	1.28	0.32-5.15	0.72	0	Fixed
Diarrhoea	5	1.25	0.82-1.90	0.29	0	Fixed
Severe AE	4	1.22	0.88-1.69	0.23	0	Fixed
Any event of special interest leading to discontinuation	2	1.16	0.56-2.41	0.69	0	Fixed
Renal event	3	1.16	0.64-2.13	0.62	0	Fixed
Severe AE	4	1.16	0.64-2.12	0.62	27	Random
AEs leading to treatment discontinuation	3	1.03	0.64-1.65	0.90	0	Fixed
Any AE	5	1.03	0.94-1.11	0.56	34	Random
Positively of Hypoglycaemia	3	1.02	0.18-5.84	0.98	0	Fixed
Urinary tract infection	5	1.02	0.71-1.46	0.91	0	Fixed
Any SMBG value #3.0 mmol/L (#55 mg/dL)	3	1.01	0.97-1.05	0.78	0	Fixed
Vulvovaginal events	2	1.01	0.14-7.15	0.99	0	Fixed
Diarrhea of Any event of special interest leading to discontinuation	2	1.00	0.17-5.78	1.00	0	Fixed
Any of Events of special interest	3	0.99	0.98-1.01	0.24	0	Fixed
Any nocturnal documented hypoglycemia	2	0.97	0.92-1.02	0.29	0	Fixed
Documented (SMBG ≤ 70 mg/dL) of Hypoglycaemia	3	0.95	0.81-1.11	0.54	79	Random
Severe nocturnal hypoglycemia event	2	0.86	0.39-1.89	0.70	0	Fixed
Bone fracture	4	0.74	0.39-1.38	0.34	0	Fixed
Deaths	4	0.44	0.14-1.41	0.17	0	Fixed
Stroke	3	0.33	0.05-2.10	0.24	0	Fixed

### The effect of sotagliflozin on related-to-acidosis events

**Figure 3:**
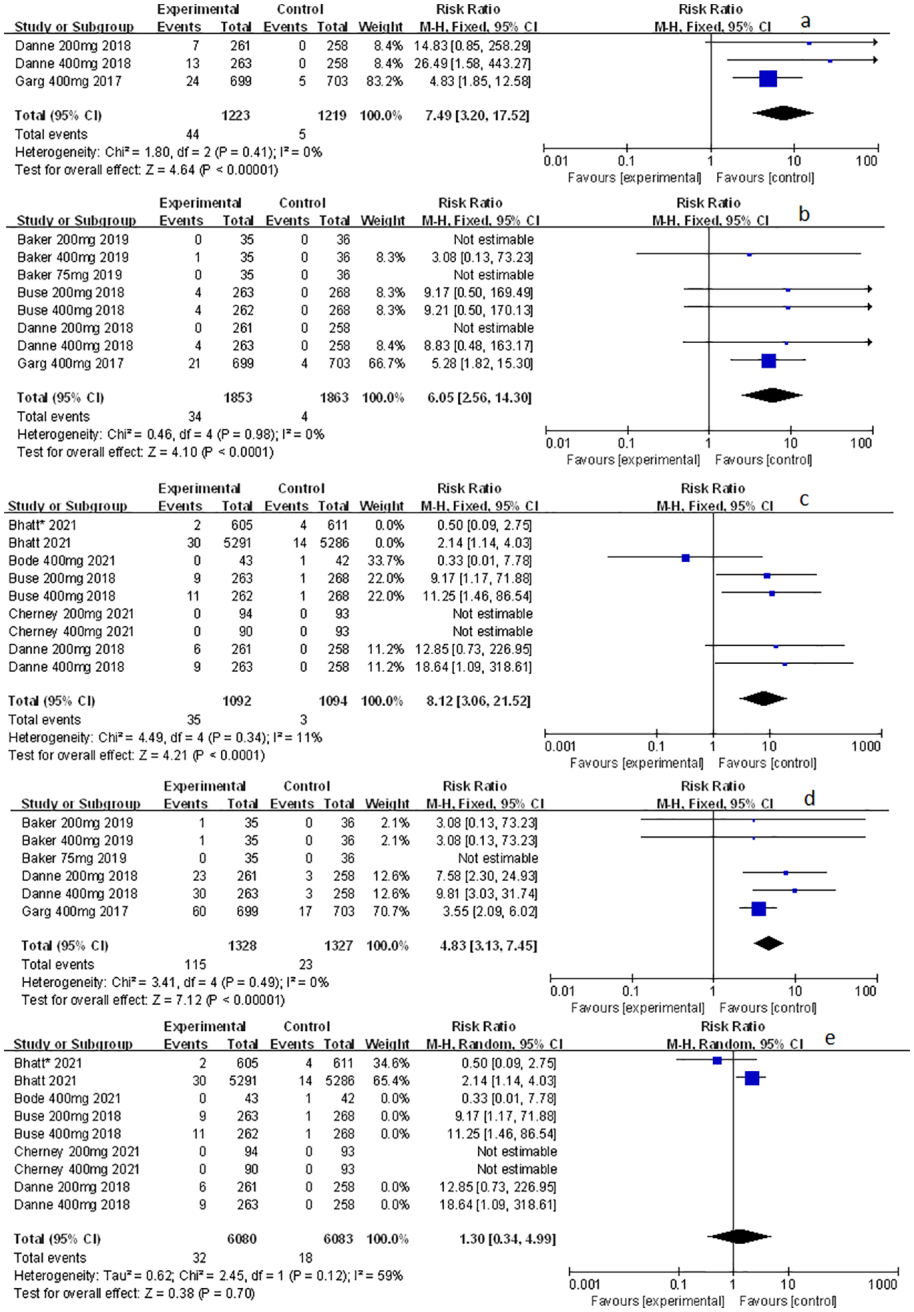
Forest plot of related to acidosis events for sotagliflozin versus the control group in T1D and T2D. **(A)** The adverse events of severe acidosis-related adverse events in T1D. **(B)** The adverse events of positively adjudicated DKA in T1D. **(C)** The adverse events of DKA in T1D. **(D)** The acidosis-related adverse events in T1D. **(E)** The adverse events of DKA in T2D.

**Figure 4:**
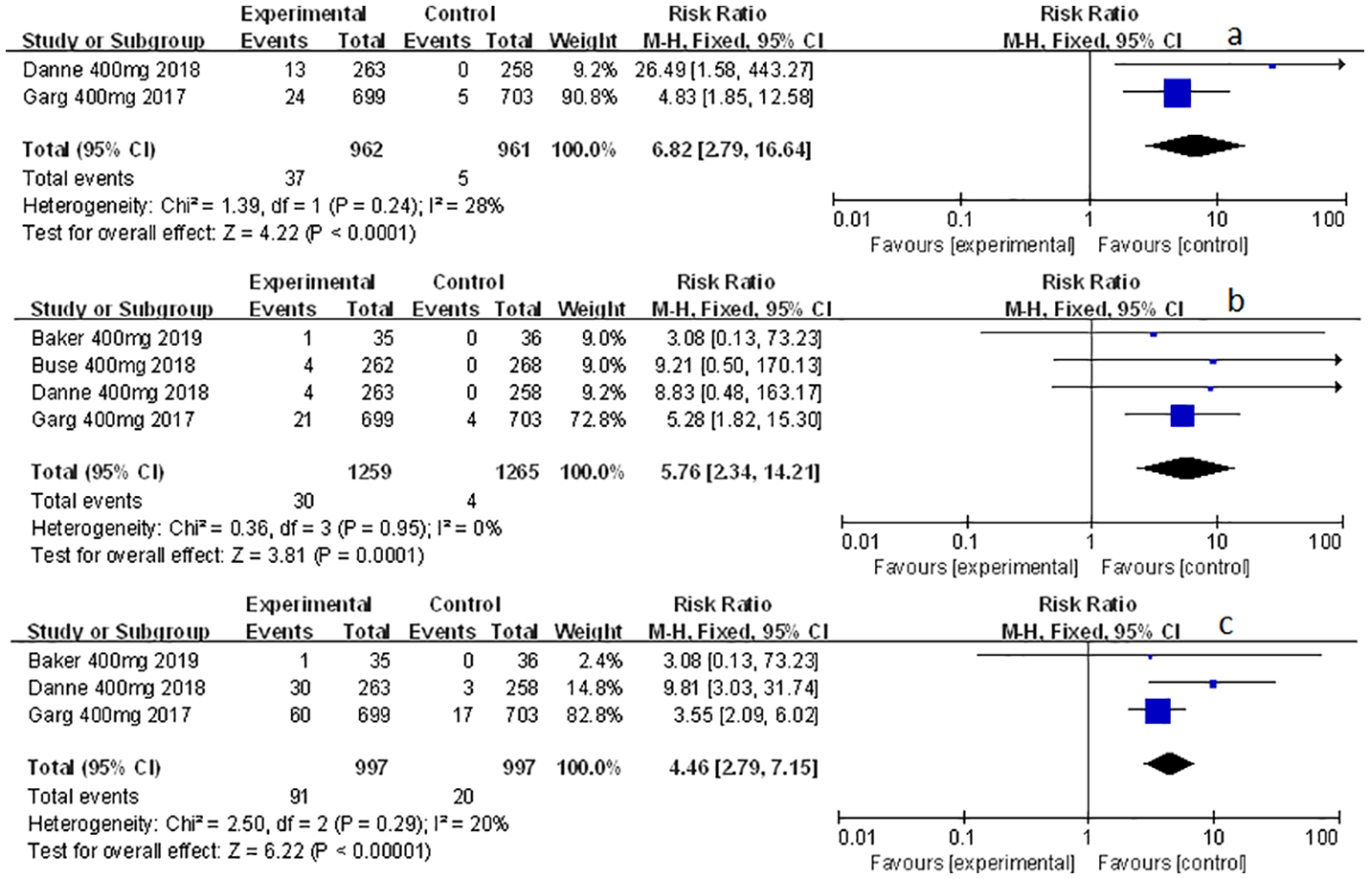
Forest plot of related to acidosis events in the subgroup of sotagliflozin 400mg versus the control group in T1D. **(A)** The adverse events of severe acidosis-related adverse events in T1D. **(B)** The adverse events of positively adjudicated DKA in T1D. **(C)** The acidosis-related adverse events in T1D.

**Figure 5:**
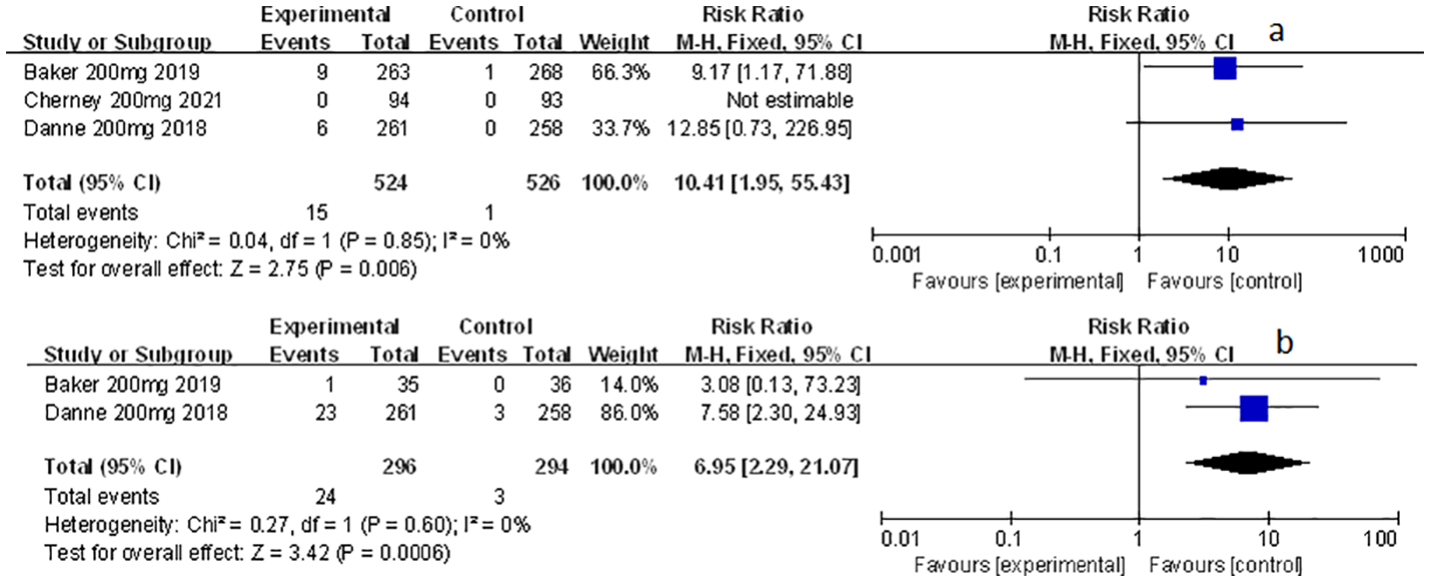
Forest plot of related to acidosis events in the subgroup of sotagliflozin 200mg versus the control group in T1D. **(A)** The adverse events of DKA in T1D. **(B)** The adverse events of acidosis-related event in T1D.

Related-to-acidosis events were mentioned in 8 articles (3, 8, 9, 15–19) in total among the selected studies. Herein, we found that sotagliflozin could increase the risk of related-to-acidosis events in the patients of T1D, such as serious of acidosis-related adverse events (RR: 7.49, 95% Cl: 3.20-17.52, *p* < 0.001; **Figure 3A**), positively adjudicated diabetic ketoacidosis (DKA) (RR: 6.05, 95% Cl: 2.56-14.30, *p* < 0.001; **Figure 3B**), DKA (RR: 8.12, 95% Cl: 3.06-21.52, *p* < 0.001; **Figure 3C**), and acidosis-related event (RR: 4.83, 95% Cl: 3.13-7.45, *p* < 0.001; **Figure 3D**). In the patients of T2D, sotagliflozin could not increase the risk of DKA (RR: 1.30, 95% Cl: 0.34-4.99, *p* = 0.70; **Figure 3E**). As for serious of acidosis-related adverse events, positively adjudicated diabetic ketoacidosis (DKA) and acidosis-related event, the included studies were not reported for T2D patients. An increase in risk of serious of acidosis-related adverse events, positively adjudicated DKA, and acidosis-related event (RR: 6.82, 95% Cl: 2.79-16.64, *p* < 0.001; **Figure 4A**; RR: 5.76, 95% Cl: 2.34-14.21, *p* < 0.001; **Figure 4B**; RR: 4.46, 95% Cl: 2.79-7.15, *p* < 0.001; **Figure 4C**; respectively) were observed in the subgroup of sotagliflozin 400 mg comparing with placebo in T1D patients. In the group of sotagliflozin 200 mg versus placebo, an increase was discovered in the risks of DKA (RR: 10.41, 95% Cl: 1.95-55.43, *p* < 0.01; **Figure 5A**), and acidosis-related event (RR: 6.95, 95% Cl: 2.29-21.07, *p* < 0.001; **Figure 5B**) in T1D patients. As for the included studies about the dosage of sotagliflozin 200mg and 400mg with patients in T2D, the serious of acidosis-related adverse events, positively adjudicated diabetic ketoacidosis (DKA), acidosis-related event and DKA were not reported.

### The effect of sotagliflozin on the other related adverse events

**Figure 6:**
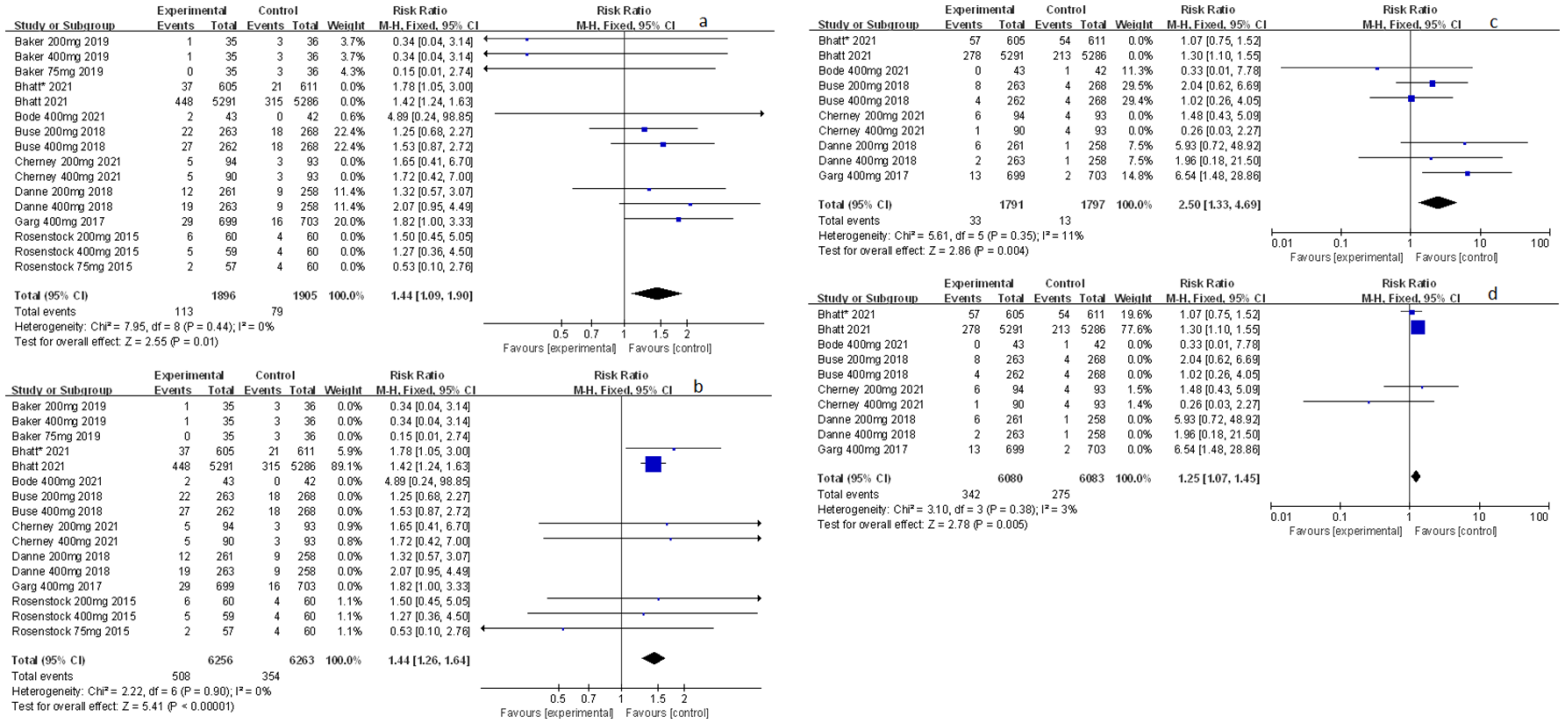
Forest plot of the other related adverse events for sotagliflozin versus the control group in T1D and T2D. **(A)** The adverse event of diarrhea in T1D. **(B)** The adverse event of diarrhea in T2D. **(C)** The adverse event of volume depletion in T1D. **(D)** The adverse event of volume depletion in T2D.

**Figure 7:**
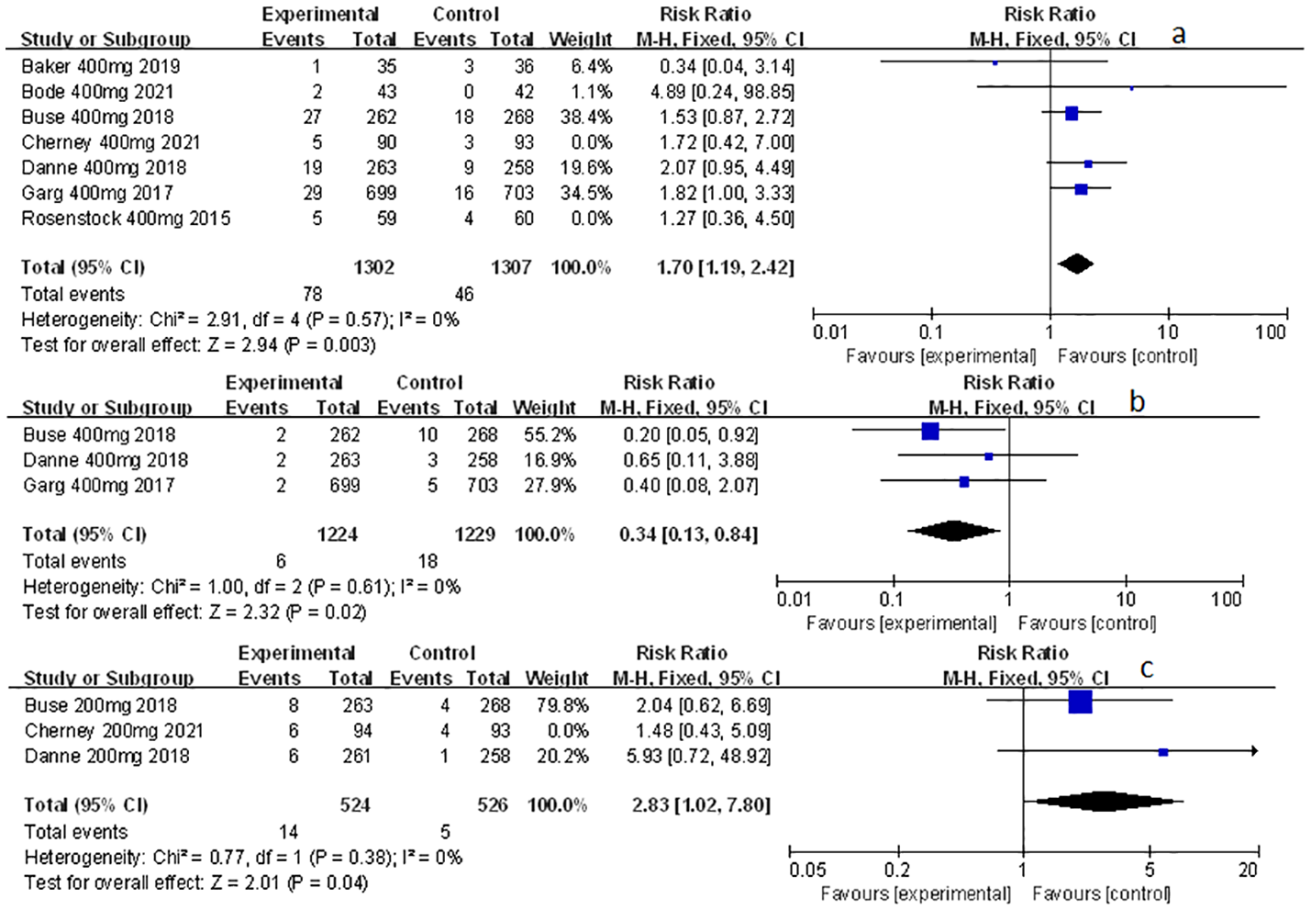
Forest plot of the other related adverse events in the subgroup of sotagliflozin 400mg and 200mg versus the control group in T1D. **(A)** The adverse event of diarrhea in sotagliflozin 400 mg versus placebo. **(B)** The adverse event of severe nocturnal hypoglycemia event in sotagliflozin 400 mg versus placebo. **(C)** The adverse event of volume depletion in sotagliflozin 200 mg versus placebo.

Other related adverse events (including diarrhea, severe nocturnal hypoglycemia event, and volume depletion) were also mentioned and analyzed in this meta-study. Among which sotagliflozin was found to be relevant to the increased risk of diarrhea and volume depletion (RR: 1.44, 95% Cl: 1.09-1.90, *p* = 0.01; **Figure 6A**; RR: 2.50, 95% Cl: 1.33-4.69, *p* < 0.01; **Figure 6C**; respectively) in patients of T1D. Also, sotagliflozin increased the risk of diarrhea and volume depletion (RR: 1.44, 95% Cl: 1.26-1.64, *p* < 0.001; **Figure 6B**; RR: 1.25, 95% Cl: 1.07-1.45, *p* < 0.01; **Figure 6D**; respectively) in patients of T2D. In the subgroup of sotagliflozin 400 mg versus placebo, sotagliflozin could also increase the risk of diarrhea (RR: 1.70, 95% Cl: 1.19-2.42, p < 0.01; **Figure 7A**), reduce the risk of severe nocturnal hypoglycemia event (RR: 0.34, 95% Cl: 0.13-0.84, *p* = 0.02; **Figure 7B**) in patients of T1D. As for sotagliflozin 200 mg versus placebo in T1D patients, sotagliflozin was found to be related to the increased risk in volume depletion (RR: 2.83, 95% Cl: 1.02-7.80; **Figure 7C**).

### The publication bias and sensitivity analysis with sotagliflozin on genital mycotic infection

In response to the relevant articles included in this study, a bias test of the relationship between sotagliflozin and genital mycotic infection was published. No publishing bias, both of *p*-value > 0.05, was discovered through the bias test. Moreover, the results of sensitivity analysis revealed the stability of sequentially removing each group (**Supplementary Figures 2**, **3**).

## Discussion

Sotagliflozin, a dual inhibitor for SGLT1 and SGLT2, can improve the level of blood glucose, control body weight, lower the level of glycated hemoglobin, and decrease the dosage of insulin in its combined therapy (6, 8, 9). Danne et al. noted that the combined therapy with sotagliflozin and insulin significantly prolonged glucose TIR (time in range), resulting in improved and controlled glycemia (21). In addition, the use of sotagliflozin increased DAOH (days alive and out of the hospital). This can lighten the patient’s burden of disease by reducing their additional cost and allowing them to have an improved quality of life (22). Apart from the aforementioned effects, sotagliflozin has also been shown to be effective in lowering systolic blood pressure, and treating heart failure and myocardial infarction (12). Rodbard et al. showed that the use of sotagliflozin significantly reduced blood pressure, several markers of arterial stiffness and vascular resistance in T1D patients (23). Notably, Bhatt et al. also pointed out that, compared to the placebo group, in diabetic patients with recently worsened heart failure (or chronic kidney disease with or without proteinuria), the use of sotagliflozin before or shortly after discharge from the hospital significantly reduced the total number of cardiovascular-related deaths, hospitalizations for heart failure and emergency visits (16, 17) in T2D patients. However, adverse events including genital infections, DKA, diarrhea, and increased volume loss are widely considered in the pharmaceutical and medical fields due to their considerable presence (8, 10, 12), which is consistent with the results here. In addition to the AEs mentioned in other articles, this analysis showed that sotagliflozin was also associated with a reduced incidence of severe nocturnal hypoglycemia events.

Moreover, sotagliflozin may significantly increase the risk of genital mycotic infection in DM patients. Lega, Engelhardt, McGill, et al. showed that an SGLT2 inhibitor (one of the API (Active pharmacological Ingredient) of sotagliflozin) can cause the incidence of genital mycotic infection in DM patients, especially in men and aged women, of which the onset of genital mycotic infection was usually within the next 30 days following the use of SGLT2 inhibitors (24–26). SGLT2 inhibitors were found to reduce glucose reabsorption in the kidney, resulting in glucosuria (27). We suspect that the change of environment may be related to the genital mycotic infection. As the concentration of sugar in the environment becomes high, Candida Albicans, the dominating pathogen in DM patients, grows rampantly and establish genital mycotic infection. This may be the possible mechanism of the relationship between sotagliflozin and the increased incidence of genital mycotic infection (28). In addition, the cultivation of Candida Albicans is also related to patients’ compromised immune function. T1D is an autoimmune disease caused by the invasion of pancreatic islets by immune cells that selectively kill β cells (29). Therefore, we speculate that, due to genital flora dysbiosis caused by autoimmune imbalance, the environmental changes caused by the use of sotagliflozin may increase an additional risk of genital mycotic infection in the treatment of T1D (30). Interestingly, Nyirjesy (28) et al. pointed out that genital mycotic infection can be effectively avoided if antifungal ointment is used as a supplementary therapy. We believe that this complementary method can be applied in addition to the normal sotagliflozin treatment to improve safety by raising fewer systemic adverse events.

As for the more acidosis-related events related to sotagliflozin, we speculate that the higher formation rate of the ketone body may be the possible mechanism (30). Studies have shown that the elevation in plasma glucagon/insulin ratio induced by SGLT2 inhibitors is the result of interference with islet cells. This may result from a metabolic reaction where SGLT2 inhibitors decrease the blood sugar and insulin levels *via* glycosuria, as well as raise the level of blood glucose accompanied by ketone production (31). In addition, the significant elevation in plasma glucose level may also be related to the newly recognized secretion of SGLT1 in α cells to regulate glucose secretion (32). In T1D treatment, Danne et al. observed more DKA records when sotagliflozin was supplemented with insulin alone (9), which limits its application. Despite the aforementioned side effects, sotagliflozin is still a promising medication that may allow more patients to achieve targeted glycemic control without increasing the risk of weight gain or hypoglycemia, especially with the aid of proper consultation and education while dispensing the prescription (33) in patients with T1D. According to existing studies in patients with T1D, despite the best insulin treatment, sotagliflozin could benefit the diabetic patients who have failed to achieve adequate blood sugar control and are at relatively low risk of DKA, who can be thoroughly informed about the related complication and who are in close contact with their doctor (34). Studies have shown that in patients with T1D, the incidence of DKA is reduced with the aid of intensive risk mitigation programs, suggesting the vital role of patients’ education played throughout the treatment process (35). At the same time, T1D patients should be instructed to be aware of dangerous situations in terms of teaching them how to measure blood ketones (36). Also, the risk of DKA can be minimized (37) by identifying right patient group and decreasing basal insulin dose in patients with T1D.

Additionally, there are diarrhea, volume depletion, and severe nocturnal hypoglycemia events related to sotagliflozin. The increased incidence of diarrhea and volume depletion (12) may be related to the inhibition of SGLT1, one of the binding sites of sotagliflozin. Danne et al. discovered that SGLT1, expressed in GI tract, is mainly responsible for the absorption and transport of glucose and galactose (2). The inhibition may cause gastrointestinal dysfunction and other reactions, as a result of which the incidence of diarrhea and subsequent volume depletion increases. In addition, Rodbard et al. (23) showed that giving sotagliflozin alongside insulin can significantly reduce blood pressure, which is suspected to be another mechanism of volume loss events.

Moreover, the combination of sotagliflozin and optimized insulin therapy was found to significantly reduce the incidence of severe hypoglycemia in patients with T1D (8, 9). It is speculated that this is related to the dosage of insulin. Danne et al. have shown that in patients with T1D, hypoglycemia is usually induced by sotagliflozin when insulin therapy is intensified (38). However, the existing studies did not elaborate on the relationship between hypoglycemia and sotagliflozin. Therefore, further trials on the relationship between insulin combined with sotagliflozin therapy and hypoglycaemia should be initiated. One promising example is to design new pharmaceutical forms based on sotagliflozin stents to treat T2D, and other types of diabetes (T2D with Alzheimer’s disease) as well (39), so that patients with DM can make full use of their medication and minimize or delay insulin use, thereby improving their quality of life.

When deciding on the dose of sotagliflozin, a strong correlation between adverse events and dose was revealed. This analysis shows that the 200-mg dosage has a relatively lower risk of general adverse events, while the 400-mg dosage is discovered to be able to significantly reduce the risk of severe nocturnal hypoglycemia events. Interestingly, Baker et al. also showed (15) that the 200-mg and 400-mg dosages improved blood sugar and maintained body weight in T1D patients. Moreover, the 400-mg dose can also lower the PPG levels, resulting in a lower incidence of severe hypoglycemia. Therefore, sotagliflozin with a dose of 400 mg is recommended here. Further clinical studies are needed however, to support the widespread use of this dose.

## Conclusions

In a word, our research shows that the adverse events of sotagliflozin are tolerable to patients with DM to a certain extent, in terms of the incidence of genital mycotic infection, related-to-acidosis events, diarrhea, volume loss, and severe nocturnal hypoglycemia events. Moreover, optimization and finer regulation of the drug should be encouraged (15). A subgroup analysis of the dose of sotagliflozin was additionally conducted and found that the use of a 400mg dose had a greater clinical significance. However, due to the small number of articles included in this study, further clinical confirmation is needed for sotaglifozin to be better applied to patients with DM.

## Strengths and shortcomings

The study was the first to comprehensively and systematically analyze the adverse events of sotagliflozin in patients with DM. All of the included clinical studies were RCT with a high level of evidence. Furthermore, the study also conducted a subgroup analysis based on the dose of sotagliflozin, which paves a vital path for later clinical use. However, there are still several shortcomings: 1. Since sotagliflozin is not yet marketed, there are only a limited number of randomized controlled trials related to it, which was too small to drastically analyze the adverse events stratified by the diagnosis of T1D and T2D; 2. About the dosage of sotagliflozin, the 400 mg needs to be supported by further clinical studies; 3. In the aspect of SGLT-inhibition type, there are barely relevant randomized controlled trials about the comparative studies of sotagliflozin with dapagliflozin, empagliflozin, canagliflozin, ertugliflozin to identify the effects of additional SGLT1-inhibition. Therefore, the conclusions of this meta-analysis need to be further validated in clinical practices.

## Data availability statement

The original contributions presented in the study are included in the article/**Supplementary Material**. Further inquiries can be directed to the corresponding author.

## Author contributions

Authors’ contributions: all authors helped to perform the research. FZ, ND, and QS manuscript writing. LZ performed procedures and data analysis. CW contributed to writing the manuscript. HR contributed to drafting conception and design. All authors contributed to the article and approved the submitted version

## Funding

This study was supported by the National Key R&D Program of China (2022YFC3600100).

## Conflict of interest

The authors declare that the research was conducted in the absence of any commercial or financial relationships that could be construed as a potential conflict of interest.

## Publisher’s note

All claims expressed in this article are solely those of the authors and do not necessarily represent those of their affiliated organizations, or those of the publisher, the editors and the reviewers. Any product that may be evaluated in this article, or claim that may be made by its manufacturer, is not guaranteed or endorsed by the publisher.

## Supplementary material

Supplementary Figure 1 Flow diagram of study inclusion and exclusion. A total of 281 papers were retrieved from PubMed, Web of Science, EBSCO, and Cochrane libraries with the terms described in the method, among which 184 papers are research articles, but not review, or systematic review, or meta-analysis. Furthermore, retrospective studies, observational studies and experimental studies were then excluded to produce a list of 52 papers, out of which, 9 high-quality RCT studies were selected for meta-analysis in this study.

Supplementary Figure 2 Publication bias of the secondary clinical outcomes for KMC versus the control group.

Supplementary Figure 3 Sensitivity analysis of the secondary clinical outcomes for KMC versus the control group.

## Abbreviations

DM, Diabetes mellitus; SGLT1, sodium-glucose cotransporter 1; SGLT2, sodium-glucose cotransporter 2; T1D, type 1 diabetes; T2D, type 2 diabetes; BMI, body mass index; DKA, diabetic ketoacidosis; RCTs, randomized controlled trials; RR, risk ratio; 95%CI, confidence interval of 95%.

## References

[1] Bhattacharya S Rathore A Parwani D Mallick C Asati V Agarwal S . An exhaustive perspective on structural insights of SGLT2 inhibitors: A novel class of antidiabetic agent. Eur J Med Chem (2020) 204:112523. doi: 10.1016/j.ejmech.2020.112523 32717480

[2] Danne T Biester T Kordonouri O . Combined SGLT1 and SGLT2 inhibitors and their role in diabetes care. Diabetes Technol Ther (2018) 20(S2):S269–77. doi: 10.1089/dia.2018.0081 29916741

[3] Garg SK Henry RR Banks P Buse JB Davies MJ Fulcher GR . Effects of sotagliflozin added to insulin in patients with type 1 diabetes. N Engl J Med (2017) 377(24):2337–48. doi: 10.1056/NEJMoa1708337 28899222

[4] Powell DR Zambrowicz B Morrow L Beysen C Hompesch M Turner S . Sotagliflozin decreases postprandial glucose and insulin concentrations by delaying intestinal glucose absorption. J Clin Endocrinol Metab (2020) 105(4):e1235–1249. doi: 10.1210/clinem/dgz258 PMC7067537 31837264

[5] Danne T Edelman S Frias JP Ampudia-Blasco FJ Banks P Jiang W . Efficacy and safety of adding sotagliflozin, a dual sodium-glucose co-transporter (SGLT)1 and SGLT2 inhibitor, to optimized insulin therapy in adults with type 1 diabetes and baseline body mass index ≥ 27 kg/m(2). Diabetes Obes Metab (2021) 23(3):854–60. doi: 10.1111/dom.14271 33289297

[6] Rodbard HW Giaccari A Lajara R Stewart J Strumph PS Oliveira J . Sotagliflozin added to optimized insulin therapy leads to HbA1c reduction without weight gain in adults with type 1 diabetes: A pooled analysis of inTandem1 and inTandem2. Diabetes Obes Metab (2020) 22(11):2089–96. doi: 10.1111/dom.14127 32618383

[7] Sands AT Zambrowicz BP Rosenstock J Lapuerta P Bode BW Garg SK . Sotagliflozin, a dual SGLT1 and SGLT2 inhibitor, as adjunct therapy to insulin in type 1 diabetes. Diabetes Care (2015) 38(7):1181–8. doi: 10.2337/dc14-2806 PMC4831906 26049551

[8] Buse JB Garg SK Rosenstock J Bailey TS Banks P Bode BW . Sotagliflozin in combination with optimized insulin therapy in adults with type 1 diabetes: The north American inTandem1 study. Diabetes Care (2018) 41(9):1970–80. doi: 10.2337/dc18-0343 PMC6105319 29937430

[9] Danne T Cariou B Banks P Brandle M Brath H Franek E . HbA(1c) and hypoglycemia reductions at 24 and 52 weeks with sotagliflozin in combination with insulin in adults with type 1 diabetes: The European inTandem2 study. Diabetes Care (2018) 41(9):1981–90. doi: 10.2337/dc18-0342 29937431

[10] Sims H Smith KH Bramlage P Minguet J . Sotagliflozin: a dual sodium-glucose co-transporter-1 and -2 inhibitor for the management of type 1 and type 2 diabetes mellitus. Diabetic medicine: J Br Diabetic Assoc (2018) 35(8):1037–48. doi: 10.1111/dme.13645 29637608

[11] Rendell MS . The journey from gene knockout to clinical medicine: telotristat and sotagliflozin. Drug Design Dev Ther (2019) 13:817–24. doi: 10.2147/DDDT.S144556 PMC6408923 30880915

[12] Avgerinos I Karagiannis T Kakotrichi P Michailidis T Liakos A Matthews DR . Sotagliflozin for patients with type 2 diabetes: A systematic review and meta-analysis. Diabetes Obes Metab (2022) 24(1):106–14. doi: 10.1111/dom.14555 34545668

[13] Gu L Du N Jin Q Li S Xie L Mo J . Magnitude of benefit of the addition of poly ADP-ribose polymerase (PARP) inhibitors to therapy for malignant tumor: A meta-analysis. Crit Rev Oncology/Hematology (2020) 147:102888. doi: 10.1016/j.critrevonc.2020.102888 32018126

[14] Du N Chen M Shen Z Li S Chen P Khadaroo PA . Comparison of quality of life and nutritional status of between roux-en-Y and billroth-I reconstruction after distal gastrectomy: A systematic review and meta-analysis. Nutr Cancer (2020) 72(5):849–57. doi: 10.1080/01635581.2019.1656262 31460799

[15] Baker C Wason S Banks P Sawhney S Chang A Danne T . Dose-dependent glycometabolic effects of sotagliflozin on type 1 diabetes over 12 weeks: The inTandem4 trial. Diabetes Obes Metab (2019) 21(11):2440–9. doi: 10.1111/dom.13825 PMC6851757 31264767

[16] Bhatt DL Szarek M Pitt B Cannon CP Leiter LA McGuire DK . Sotagliflozin in patients with diabetes and chronic kidney disease. N Engl J Med (2021) 384(2):129–39. doi: 10.1056/NEJMoa2030186 33200891

[17] Bhatt DL Szarek M Steg PG Cannon CP Leiter LA McGuire DK . Sotagliflozin in patients with diabetes and recent worsening heart failure. N Engl J Med (2021) 384(2):117–28. doi: 10.1056/NEJMoa2030183 33200892

[18] Bode BW Cengiz E Wadwa RP Banks P Danne T Kushner JA . Effects of sotagliflozin combined with intensive insulin therapy in young adults with poorly controlled type 1 diabetes: The JDRF sotagliflozin study. Diabetes Technol Ther (2021) 23(1):59–69. doi: 10.1089/dia.2020.0079 32640846 PMC7864092

[19] Cherney DZI Ferrannini E Umpierrez GE Peters AL Rosenstock J Carroll AK . Efficacy and safety of sotagliflozin in patients with type 2 diabetes and severe renal impairment. Diabetes Obes Metab (2021) 23(12):2632–42. doi: 10.1111/dom.14513 34338408

[20] Rosenstock J Cefalu WT Lapuerta P Zambrowicz B Ogbaa I Banks P . Greater dose-ranging effects on A1C levels than on glucosuria with LX4211, a dual inhibitor of SGLT1 and SGLT2, in patients with type 2 diabetes on metformin monotherapy. Diabetes Care (2015) 38(3):431–8. doi: 10.2337/dc14-0890 PMC5131876 25216510

[21] Danne T Cariou B Buse JB Garg SK Rosenstock J Banks P . Improved time in range and glycemic variability with sotagliflozin in combination with insulin in adults with type 1 diabetes: A pooled analysis of 24-week continuous glucose monitoring data from the inTandem program. Diabetes Care (2019) 42(5):919–30. doi: 10.2337/dc18-2149 PMC6905498 30833371

[22] Szarek M Bhatt DL Steg PG Cannon CP Leiter LA McGuire DK . Effect of sotagliflozin on total hospitalizations in patients with type 2 diabetes and worsening heart failure: A randomized trial. Ann Internal Med (2021) 174(8):1065–72. doi: 10.7326/M21-0651 34152828

[23] Rodbard HW Giaccari A Cariou B Garg S Davies MJ Seth K . Effect of sotagliflozin as an adjunct to insulin therapy on blood pressure and arterial stiffness in adults with type 1 diabetes: A *post hoc* pooled analysis of inTandem1 and inTandem2. Diabetes Vasc Dis Res (2021) 18(1):1479164121995928. doi: 10.1177/1479164121995928 PMC8481733 33611925

[24] Lega IC Bronskill SE Campitelli MA Guan J Stall NM Lam K . Sodium glucose cotransporter 2 inhibitors and risk of genital mycotic and urinary tract infection: A population-based study of older women and men with diabetes. Diabetes Obes Metab (2019) 21(11):2394–404. doi: 10.1111/dom.13820 31264755

[25] Engelhardt K Ferguson M Rosselli JL . Prevention and management of genital mycotic infections in the setting of sodium-glucose cotransporter 2 inhibitors. Ann Pharmacother (2021) 55(4):543–8. doi: 10.1177/1060028020951928 32808541

[26] McGill JB Subramanian S . Safety of sodium-glucose Co-transporter 2 inhibitors. Am J Cardiol (2019) 124(Suppl 1):S45–s52. doi: 10.1016/j.amjcard.2019.10.029 31741440

[27] Arakaki RF . Sodium-glucose cotransporter-2 inhibitors and genital and urinary tract infections in type 2 diabetes. Postgraduate Med (2016) 128(4):409–17. doi: 10.1080/00325481.2016.1167570 26982554

[28] Nyirjesy P Sobel JD . Genital mycotic infections in patients with diabetes. Postgraduate Med (2013) 125(3):33–46. doi: 10.3810/pgm.2013.05.2650 23748505

[29] Guay C Kruit JK Rome S Menoud V Mulder NL Jurdzinski A . Lymphocyte-derived exosomal MicroRNAs promote pancreatic β cell death and may contribute to type 1 diabetes development. Cell Metab (2019) 29(2):348–361.e346. doi: 10.1016/j.cmet.2018.09.011 30318337

[30] Nuffer W Williams B Trujillo JM . A review of sotagliflozin for use in type 1 diabetes. Ther Adv Endocrinol Metab (2019) 10:2042018819890527. doi: 10.1177/2042018819890527 31807264 PMC6880037

[31] Chae H Augustin R Gatineau E Mayoux E Bensellam M Antoine N . SGLT2 is not expressed in pancreatic α- and β-cells, and its inhibition does not directly affect glucagon and insulin secretion in rodents and humans. Mol Metab (2020) 42:101071. doi: 10.1016/j.molmet.2020.101071 32896668 PMC7554656

[32] Suga T Kikuchi O Kobayashi M Matsui S Yokota-Hashimoto H Wada E . SGLT1 in pancreatic α cells regulates glucagon secretion in mice, possibly explaining the distinct effects of SGLT2 inhibitors on plasma glucagon levels. Mol Metab (2019) 19:1–12. doi: 10.1016/j.molmet.2018.10.009 30416006 PMC6323192

[33] Akturk HK Rewers A Garg SK . SGLT inhibition: a possible adjunctive treatment for type 1 diabetes. Curr Opin Endocrinol Diabetes Obes (2018) 25(4):246–50. doi: 10.1097/MED.0000000000000423 29794497

[34] Chatzopoulos G Tziomalos K . An up-to-date evaluation of sotagliflozin for the treatment of type 1 diabetes. Expert Opin Pharmacother (2020) 21(15):1799–803. doi: 10.1080/14656566.2020.1793961 33108240

[35] Peters AL McGuire DK Danne T Kushner JA Rodbard HW Dhatariya K . Diabetic ketoacidosis and related events with sotagliflozin added to insulin in adults with type 1 diabetes: A pooled analysis of the inTandem 1 and 2 studies. Diabetes Care (2020) 43(11):2713–20. doi: 10.2337/dc20-0924 PMC7576419 32928957

[36] Sjöholm Å . Using adjuvant pharmacotherapy in the treatment of type 1 diabetes. Expert Opin Pharmacother (2021) 22(16):2143–8. doi: 10.1080/14656566.2021.1939679 34132620

[37] Musso G Gambino R Cassader M Paschetta E . Efficacy and safety of dual SGLT 1/2 inhibitor sotagliflozin in type 1 diabetes: meta-analysis of randomised controlled trials. BMJ (Clinical Res ed) (2019) 365:l1328. doi: 10.1136/bmj.l1328 PMC6454301 30967375

[38] Danne T Pettus J Giaccari A Cariou B Rodbard H Weinzimer SA . Sotagliflozin added to optimized insulin therapy leads to lower rates of clinically relevant hypoglycemic events at any HbA1c at 52 weeks in adults with type 1 diabetes. Diabetes Technol Ther (2019) 21(9):471–7. doi: 10.1089/dia.2019.0157 PMC6708262 31335194

[39] Shakil S . Molecular interaction of anti-diabetic drugs with acetylcholinesterase and sodium glucose Co-transporter 2. J Cell Biochem (2017) 118(11):3855–65. doi: 10.1002/jcb.26036 28387957

