# Supplementary figures and images for: The safety of sotagliflozin in the therapy of diabetes mellitus type 1 and type 2: A meta-analysis of randomized trials
Source: Front Endocrinol (Lausanne). 2022 Sep 26;13:968478. doi: 10.3389/fendo.2022.968478 (PMC9548998; doi:10.3389/fendo.2022.968478)

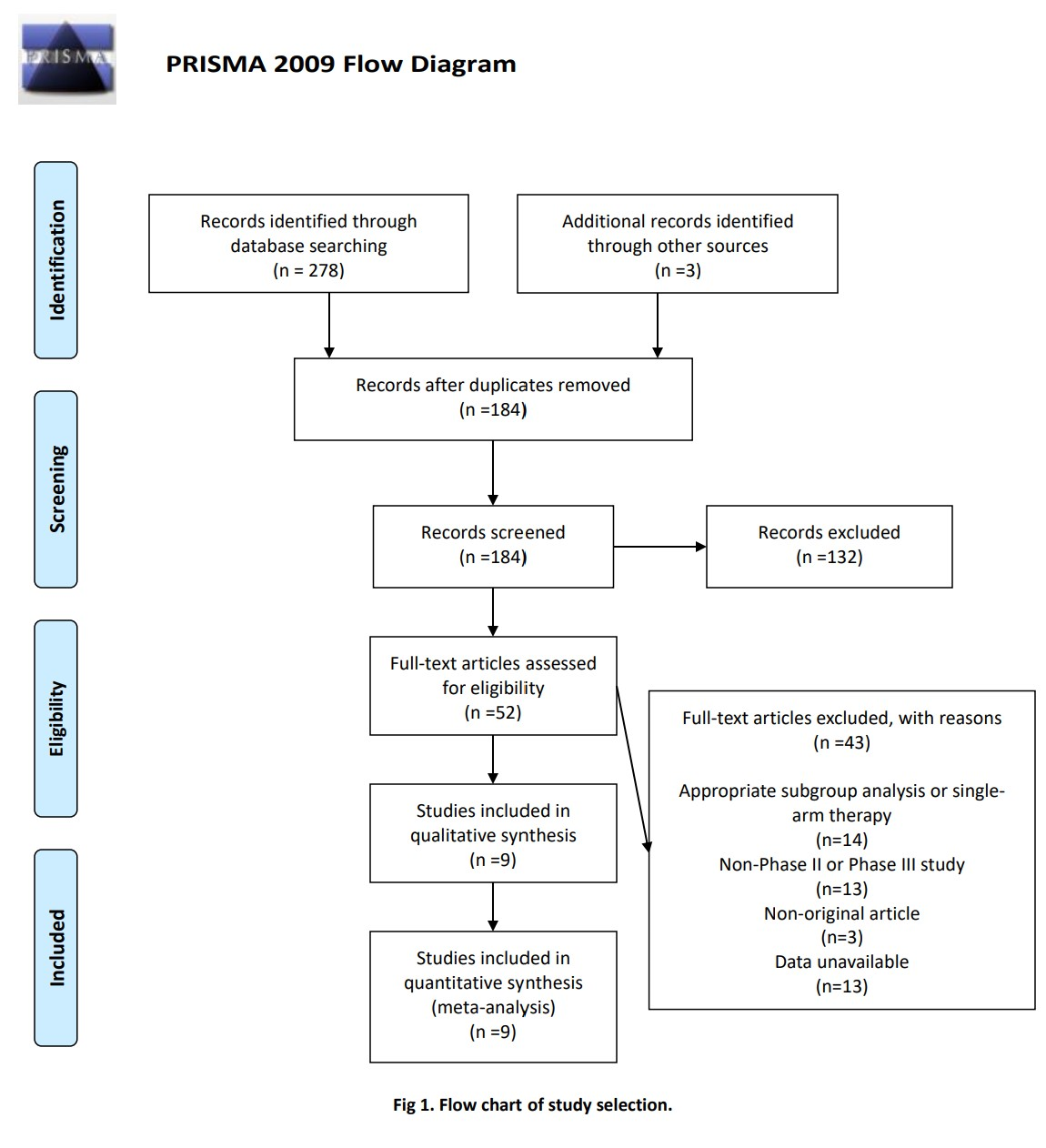

Supplement: Supplementary Figure 1 — Flow diagram of study inclusion and exclusion. A total of 281 papers were retrieved from PubMed, Web of Science, EBSCO, and Cochrane libraries with the terms described in the method, among which 184 papers are research articles, but not review, or systematic review, or meta-analysis. Furthermore, retrospective studies, observational studies and experimental studies were then excluded to produce a list of 52 papers, out of which, 9 high-quality RCT studies were selected for meta-analysis in this study. [file Image_1.tif]

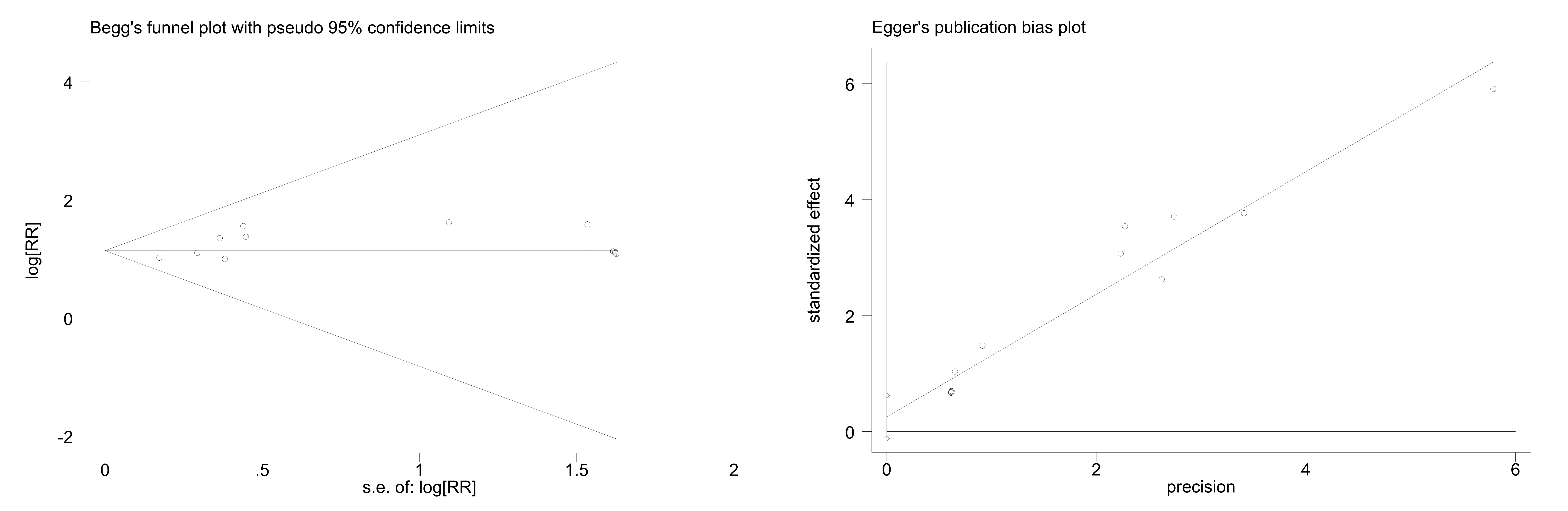

Supplement: Supplementary Figure 2 — Publication bias of the secondary clinical outcomes for KMC versus the control group. [file Image_2.tif]

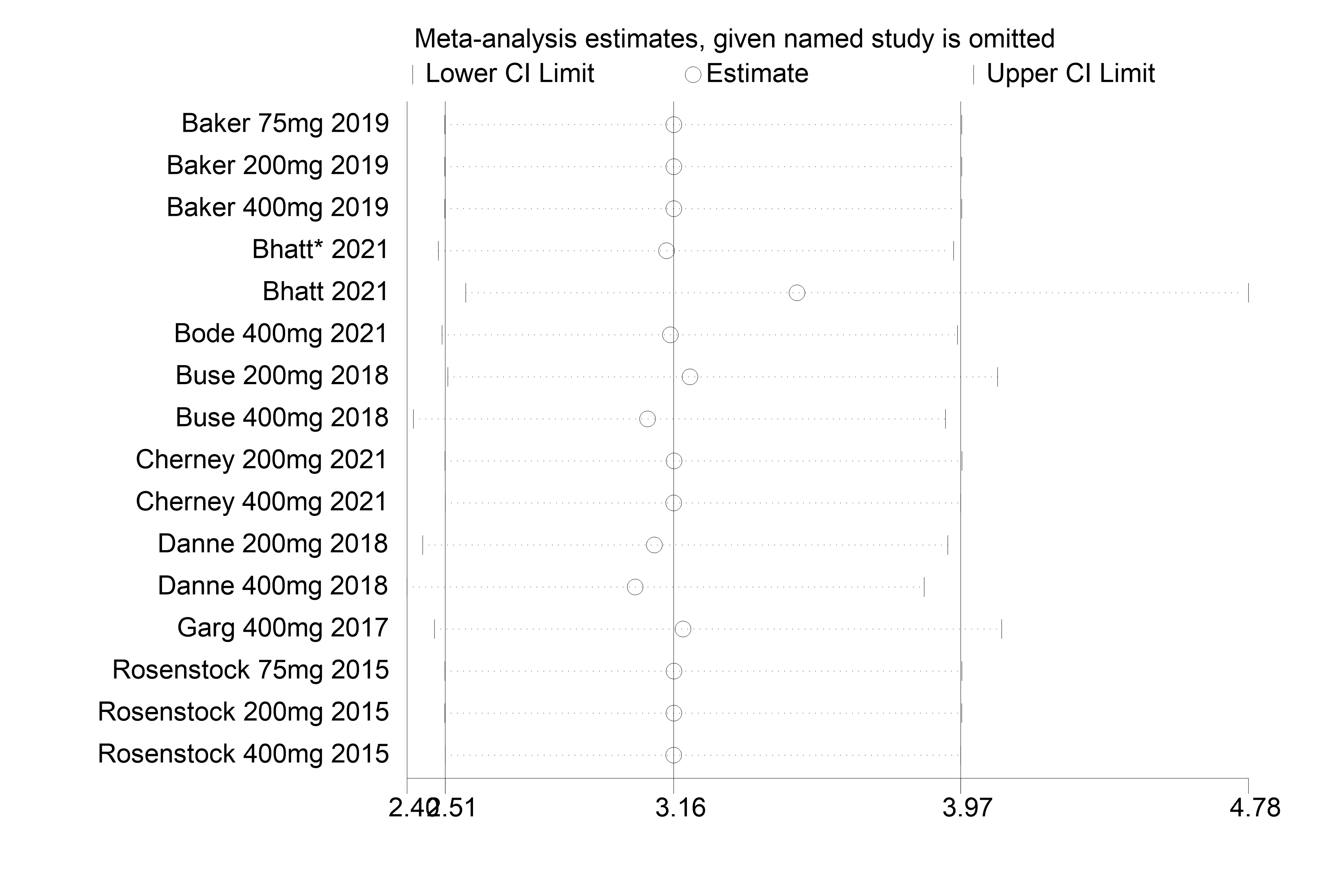

Supplement: Supplementary Figure 3 — Sensitivity analysis of the secondary clinical outcomes for KMC versus the control group. [file Image_3.tif]
