# Supplementary material for: The safety of sotagliflozin in the therapy of diabetes mellitus type 1 and type 2: A meta-analysis of randomized trials
Source: Front Endocrinol (Lausanne). 2022 Sep 26;13:968478. doi: 10.3389/fendo.2022.968478 (PMC9548998; doi:10.3389/fendo.2022.968478)
Supplement: Supplementary file 4 [file Table_1.docx]

**Supplementary Table 1**. Characteristics of included clinical trials in the meta-analysis

| Author, year | Type of study | Phase | NCT | Therapeutic regimen | | Number of inclusions | | Population of study | patient-treated years |
| --- | --- | --- | --- | --- | --- | --- | --- | --- | --- |
|  |  |  |  |  | |  | |  |  |
|  |  |  |  | Treatment | Control | Treatment | Control |  |  |
| Baker, 2019 | RCT | 2 | NCT02459899 | Sotagliflozin 75mg, 200mg, 400mg | Placebo | 105 | 36 | Type 1 diabetes | 12 weeks |
| Bhatt, 2021 | RCT | 3 | NCT03315143 | Sotagliflozin | Placebo | 5292 | 5292 | Type 2 diabetes | 22 weeks |
| Bhatt, 2021* | RCT | 3 | NCT03521934 | Sotagliflozin | Placebo | 605 | 611 | Type 2 diabetes | 22 weeks |
| Bode, 2021 | RCT | 2 | NCT02383940 | Sotagliflozin 400mg | Placebo | 43 | 42 | Type 1 diabetes | 12 weeks |
| Buse, 2018 | RCT | 3 | NCT02384941 | Sotagliflozin 200mg, 400mg | Placebo | 525 | 268 | Type 1 diabetes | 52 weeks |
| Cherney, 2021 | RCT | 3 | NCT03242018 | Sotagliflozin 200mg, 400mg | Placebo | 184 | 93 | Type 2 diabetes | 52 weeks |
| Danne, 2018 | RCT | 3 | NCT02421510 | Sotagliflozin 200mg, 400mg | Placebo | 524 | 258 | Type 1 diabetes | 52 weeks |
| Garg, 2017 | RCT | 3 | NCT02531035 | Sotagliflozin 400mg | Placebo | 699 | 703 | Type 1 diabetes | 24 weeks |
| Rosenstock, 2015 | RCT | 3 | NCT01376557 | LX4211 75mg, 200mg, 400 mg | Placebo | 179 | 60 | Type 2 diabetes | 12 weeks |

LX4211, a dual SGLT1 and SGLT2 inhibitors (Sotagliflozin). “_*_” indicated that it was not the same study.
