# Supplementary material for: The safety of sotagliflozin in the therapy of diabetes mellitus type 1 and type 2: A meta-analysis of randomized trials
Source: Front Endocrinol (Lausanne). 2022 Sep 26;13:968478. doi: 10.3389/fendo.2022.968478 (PMC9548998; doi:10.3389/fendo.2022.968478)
Supplement: Supplementary file 5 [file Table_2.docx]

**Supplementary Table 2.** **Risk of bias assessment for each of the included randomized controlled trials.**

| Author, year,  Study (RCT) | Sequence Generation | Allocation Concealment | Blinding | Incomplete outcome data | Selective outcome reporting | Free of other bias |
| --- | --- | --- | --- | --- | --- | --- |
| Baker, 2019 | low risk | low risk | low risk | low risk | low risk | low risk |
| Bhatt, 2021 | low risk | low risk | low risk | unclear risk | low risk | low risk |
| Bhatt, 2021* | low risk | low risk | low risk | low risk | low risk | low risk |
| Bode, 2021 | unclear risk | low risk | low risk | low risk | low risk | low risk |
| Buse, 2018 | low risk | low risk | low risk | low risk | low risk | low risk |
| Cherney, 2021 | low risk | low risk | low risk | low risk | low risk | low risk |
| Danne, 2018 | low risk | low risk | low risk | low risk | low risk | low risk |
| Garg, 2017 | low risk | low risk | low risk | unclear risk | low risk | low risk |
| Rosenstock, 2015 | low risk | unclear rusk | low risk | low risk | low risk | low risk |

The RCTs were assessed by the Cochrane Collaboration’s tool. Risk of bias was assessed as “low risk”, “high risk” or “unclear risk”. “_*_” indicated that it was not the same study.
